# Micelles Structure Development as a Strategy to Improve Smart Cancer Therapy

**DOI:** 10.3390/cancers10070238

**Published:** 2018-07-20

**Authors:** Nemany A. N. Hanafy, Maged El-Kemary, Stefano Leporatti

**Affiliations:** 1 Sohag Cancer Center, Sohag 82511, Egypt; nemany.hanafy@nanotec.cnr.it; 2 Institute of Nanoscience and Nanotechnology, Kafrelsheikh University, Kafrelsheikh 33516, Egypt; elkemary@sci.kfs.edu.eg; 3 CNR NANOTEC-Istituto di Nanotecnologia, 73100 Lecce, Italy

**Keywords:** micelles, hybrid polymeric and stimuli-responsive nanomicelles, cancer therapy

## Abstract

Micelles as colloidal suspension have attracted considerable attention due to their potential use for both cancer diagnosis and therapy. These structures have proven their ability to deliver poorly water-soluble anticancer drugs, improve drug stability, and have good penetration and site-specificity, leading to enhance therapeutic efficacy. Micelles are composed of hydrophobic and hydrophilic components assembled into nanosized spherical, ellipsoid, cylindrical, or unilamellar structures. For their simple formation, they are widely studied, either by using opposite polymers attachment consisting of two or more block copolymers, or by using fatty acid molecules that can modify themselves in a rounded shape. Recently, hybrid and responsive stimuli nanomicelles are formed either by integration with metal nanoparticles such as silver, gold, iron oxide nanoparticles inside micelles or by a combination of lipids and polymers into single composite. Herein, through this special issue, an updated overview of micelles development and their application for cancer therapy will be discussed.

## 1. Introduction

### 1.1. Identification of Micelles

**Scheme 1:**
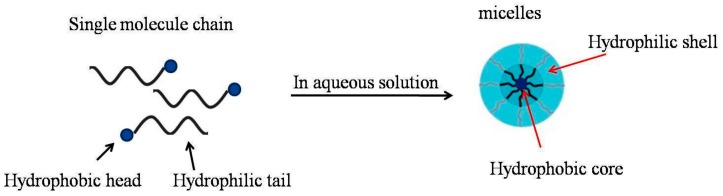
Assembled micelles structure.

Micelles are assembled colloidal dispersions having a small diameter, normally ranging from 5 to 100 nm [1,2], depending on the type of head groups and length of the alkyl chains [3,4]. Their surfactant molecules can be aggregated either by cationic, anionic, zwitterionic or non-ionic groups [5]. In aqueous solution, the non-polar hydrocarbon chain “tail” can be arranged into the center of a ball like structure “head” to form a micelle, because they are hydrophobic or “water hating” (Scheme 1) [6,7]. They can be formed from a fatty acid, a salt of a fatty acid (soap), phospholipids, or other similar molecules. In this case, micelles made with a lipid might have lower Critical Micelle Concentration (CMC) [8]. Hence, in an amphiphilic copolymer, fatty acyl chains play a valuable role as hydrophobic segments. The structure of Distearoylphosphatidyl ethanolamine (DSPE) has been used as the hydrophobic compound in a di-block copolymer with hydrophilic polyethylene oxide (PEO) to form 22 nm micelles [9]. Due to the limitation of these molecules, amphiphilic copolymers have been developed as alternative amphiphilic materials [10].

In micelle structure formation, the interaction between the polar head groups and surrounding water might cause separation between hydrophobic and hydrophilic regimens. This results in flexible and porous micelles [11]. In this case, micelles are considered a suitable model for biological applications and drug delivery systems [12], because they can increase drug solubility, reduce toxicity, prolong circulation time, enhance tissue penetration, and have targeting ability.

### 1.2. Micelles Structure

**Scheme 2:**
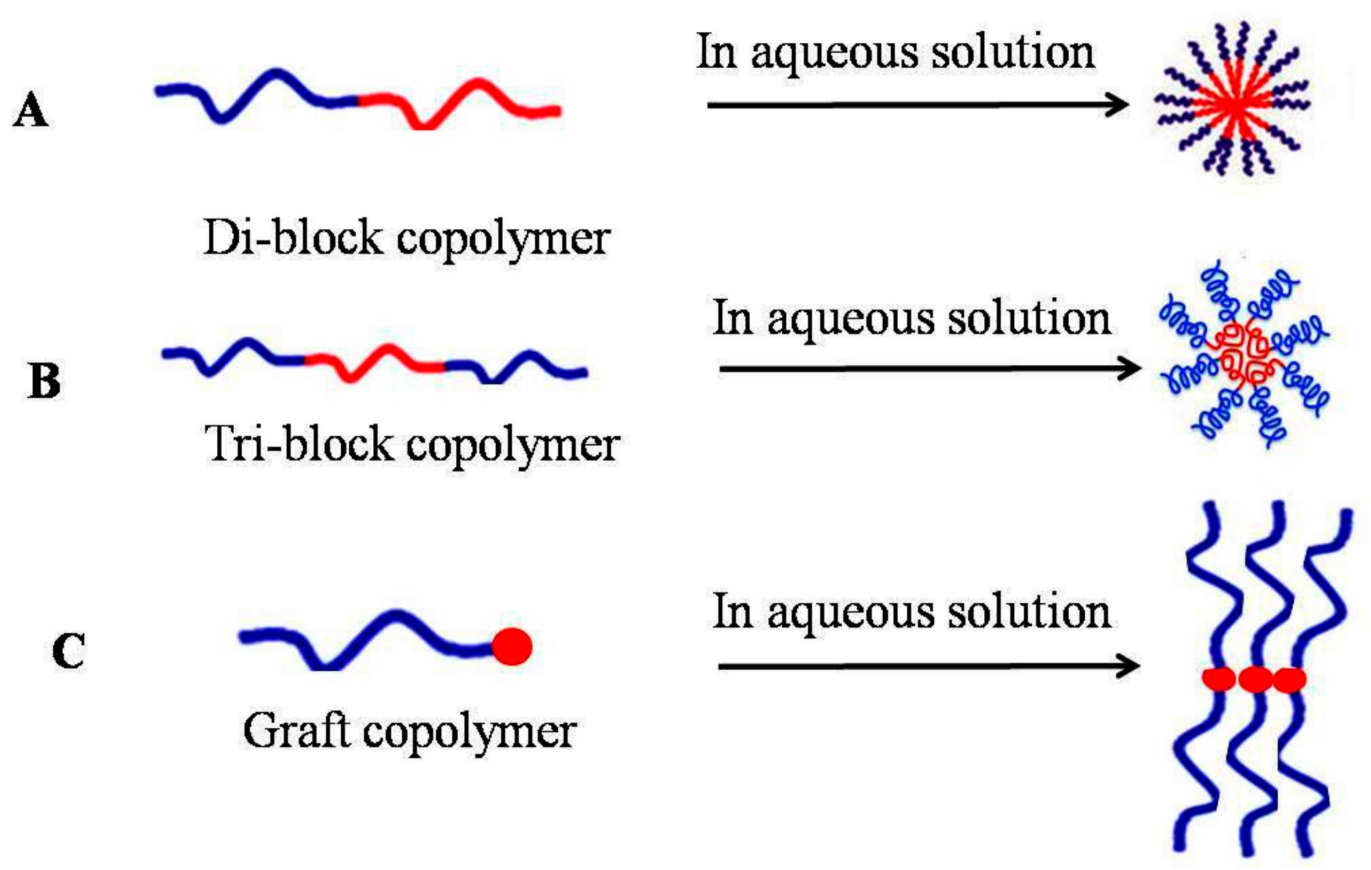
Polymer micelle structures.

Micelles are mostly composed of amphiphilic molecules in aqueous solution that self-assemble into a structure containing both hydrophobic and a hydrophilic segments (Scheme 2) [13,14,15]. In the case of concentration reduction, the amphiphiles are present as small units (monomers) in true solution, while at a high concentration, aggregation and self-assembly occur, leading to micelles formation [2]. The critical concentration that is needed to form micelles is called Critical Micelle Concentration (CMC). The micelles formed at above their CMC are being driven by dehydration of the hydrophobic tails, forming a state of entropy. Additionally, the micelles’ core will be formed by Van der Waals bonds recognition [2]. At the final structure, the hydrophilic shell forms hydrogen bond crosslink with water surrounding its out-surface [16]. Micelles can be assembled in different morphologies, such as spheres, rods, tubules, lamellae, and vesicles, depending on the quality of solvent, length of blocker chain, nature of the blocker and temperature [17,18,19]. In previous reports, the structure of micelles had been studied using numerous experimental techniques, such as nuclear magnetic resonance (NMR) [1], diffraction of X-ray (XRD) [20], photon correction spectroscopy (PCS) [21], fluorescence spectroscopy [22], electron spin resonance (ESR) [23], neutron scattering [24] and others [25].

These structures usually observe less CMC compared to low-molecular-weight surfactants. It is found that for the surfactant of low molecular weight, the CMC can be considered as 10^−3^ to 10^−4^ M, while it is 10^−6^ to 10^−7^ M for polymeric micelles. The stability of micelles’ structure remains valuable at very low polymer concentrations due to the low CMC, which makes them relatively insensitive to dilution, leading to enhance their circulation in the blood stream compared to surfactant micelles [26]. Block copolymers, also known as mosaic copolymer, is a special polymer that linked two or more polymer segments on the main chain directly [27]. Block co-polymers can be classified through the number of blocks and their arrangement [28]. Block copolymers containing two, three and more blockers are called di-blocks (AB type copolymers), tri-blocks (ABA type copolymer), and grafted copolymers respectively. Some topology can be in a linear state, in which the blocks are attached at the both ends, and stars, where blocks are linked across one of their ends at a single connection. Many attachments like brushes, (4-)miktoarm stars, or H-shape are also possible [29]. For instance, di-block co polymer (AB) can be obtained from mono-functional polymers such as photo polymerization of styrene (St) with benzyl *N*,*N*-diethyldithiocarbamate (BDC) [30], while triblock co-polymers can be synthesized from di functional polymers (e.g., *p*-xylylenebis(*N*,*N*-diethyldithiocarbamate)) [31]. For instance, poly(ethylene glycol)-*block*-poly(d,l-lactic acid) (PEG-*b*-PLLA) micelles have been extensively studied and the in vitro release of the hydrophobic drug quercetin from these micelles was investigated [32].

Grafted polymers are polymers which have branches formed from one hydrophilic backbone and one to multiple hydrophobic polymer side chains, or vice versa [33]. Drugs can be released through cellulose graft polymers. Like this, the cellulose portion can form the hydrophilic part, with any hydrophobic segment conjugated to it, resulting in an amphiphilic graft polymer. Such polymers have biodegradable properties. Hence, prednisone acetate has been delivered by Cellulose-g-poly-l-lactic acid (PLLA). Similarly, camptothecin was also released gradually from graft polymer micelles of pthaloyl chitosan and mPEG-2000 for 96 h. The diameter of amphiphilic block copolymers composites into spherical core-shell micelles reached approximately 10 to 80 nm, consisting of a hydrophobic core for drug loading. Besides, a physical barrier will be formed by a hydrophilic shell at both micelle complication in aqueous solution and to protein attachment and opsonizing state during intravenous administration [34].

Many polymers can be used to fabricate micelles. However, the selection is limited for those polymers having biocompatible and biodegradable properties, and they are either hydrophobic or hydrophilic dissolution. Steric stability of micelles is provided mainly by hydrophilic characterization of the shell, and once properly selected avoids rapid uptake by the reticulo-endothelial system (RES), leading to increase time circulating in the body [26]. Poly(ethylene glycol) (PEG) is hydrophilic polymer, having highly hydrated, an efficient steric protector, and biocompatible, with low toxicity [2,35]. Other hydrophilic polymers also can be used such as poly(*N*-vinyl pyrrolidone) (PVP) and poly(*N*-isopropylacrylamide) pNIPAM [36]. While for the hydrophobic core, polyesters, polyethers, and polyaminoacids are commonly used [35,37]. In the case of core-forming structures, many polymers are used, such as poly(propylene oxide) (PPO), poly(d,l-lactic acid) (PDLLA), poly(ε-caprolactone) (PCL), poly(l-aspartate) and poloxamers [38]. Micelles are intrinsically stealth particles when formed with a hydrophilic outer shell, and they are able to avoid engulfment by immune-system cells without further modification. Recently, block copolymers were produced by several techniques with well-defined composition, molecular weight, and structures such as single-electron-transfer living radical polymerization (SET-LRP), fragmentation chain transfer (RAFT), atom transfer radical polymerization (ATRP) and nitroxide-mediated polymerization (NMP) [39,40,41]. These combinations exhibit many positive features, such as the ability to control the composition and molecular weight of the block copolymers prepared through such methodologies.

## 2. Hybrid Polymeric Micelles

**Scheme 3:**
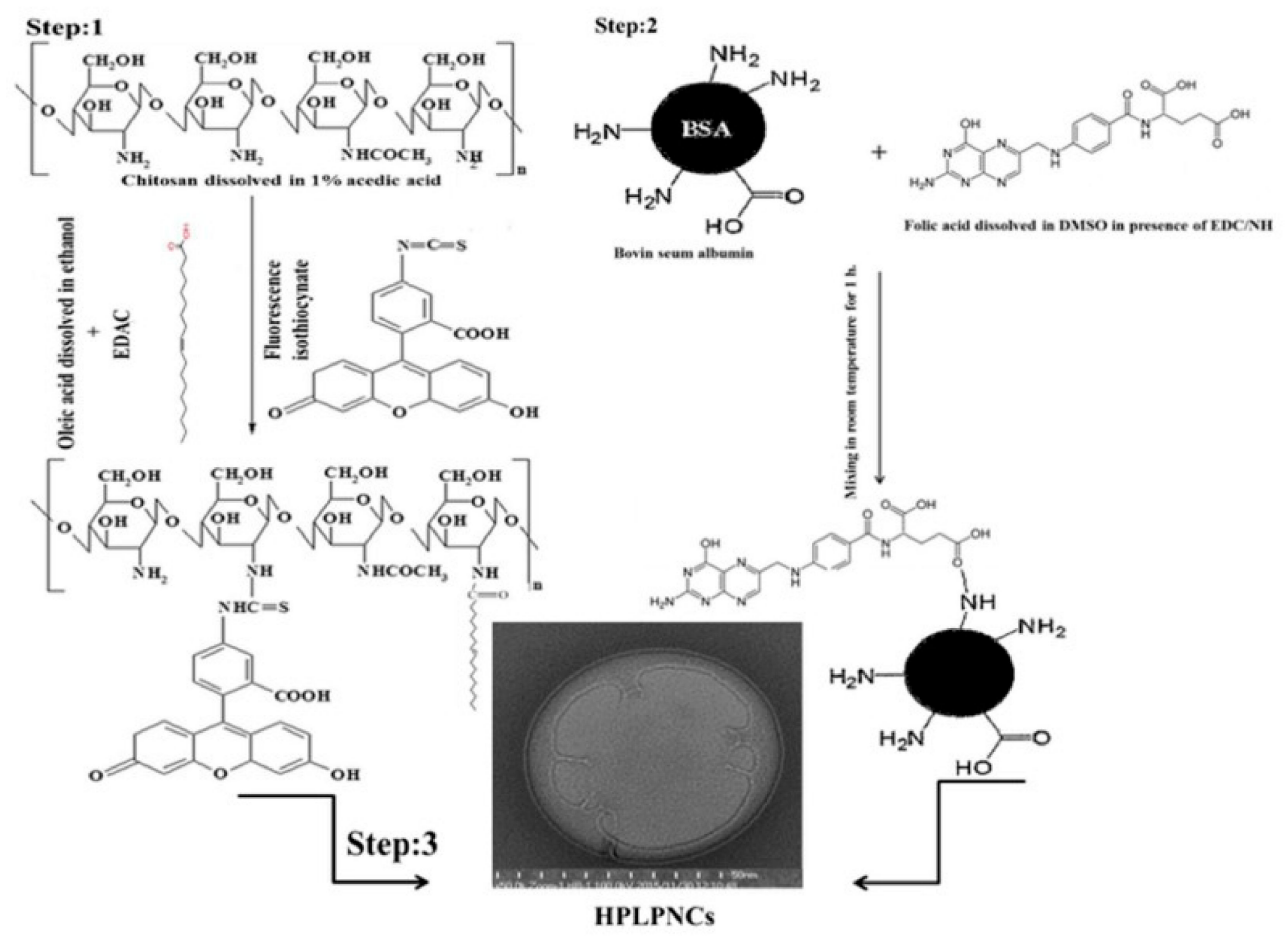
Hybrid polymer lipid protein nanocarrier. Step 1: Assembly of chitosan and oleic acid; step 2: Folic acid (FA) conjugation with bovine serum albumin; and step 3: functionalization of chitosan grafted oleic acid surface by using bovine serum albumin (BSA)-FA (Figure adapted with permission from ref. [53]).

Using polymers for directed assembly with biological molecules such as lipids, proteins, peptides and nucleotides is a very interesting approach, due to the wide range of polymers’ synthesis. Currently, there are various types of polymers available, each one has special properties and responsiveness. The polymers that have charged electrons are very useful because the biological molecules are already charged and their electrostatic interactions are a very convenient approach for assembly. Additionally, this assembly can be characterized according to hydrophobicity of polymers. The assembly of amphiphilic polymers results in formation of Nanomicelles (NMs) with a hydrophobic core and a hydrophilic shell [42]. The core-shell composite of NMs allows them to: (1) encapsulate and carry poorly water-soluble drugs; (2) decrease the bio-fouling of the NMs resulting in long circulation half-life; (3) release drugs at a sustained rate in the optimal range of drug concentration; and (4) be further functionalized with targeting ligands for differential delivery [43]. The attention was taken for micelles formed by conjugation of polyethylene glycol (PEG) and diacyl-lipids [44]. Phospholipid residues attached to PEG moieties represent short, however, extremely hydrophobic blocks due to the presence of two long-chain fatty acyl groups, and effectively form a hydrophobic core of the micelle [45]. Oleic acid (OA), as a mono-unsaturated fatty acid, was attached with chitosan by amide linkage bonds through the EDAC-mediated reaction with various degrees of amino substitution (DS), as described in a previous study [46]. Scientifically, chitosan molecules present no amphiphilic properties and, therefore, cannot form micelles in water. However, modification of chitosan chains was done by oleic acid. Hence in the presence of water-soluble carbodiimide, carboxyl groups of fatty acids were activated and ester intermediates were created (Scheme 3). Consequently, the intermediates can react with primary amine groups of chitosan to create an amide bond. The final product of this assembly is a nano-sized self-aggregation in aqueous media [47]. These rounded shape-like nuclei were furthermore coated by folic acid conjugated with bovine serum albumin (BSA) to target cancer cells and to minimize side effects [48]. Oleic acid modified chitosan caused well dispersion for micelles in aqueous media, raised amide linkage, and formed a denser hydrophobic core [49].

The lipid core micelles were first produced by composition of polyethylene glycol-phosphatidylethanolamine (PEG-PE) forming micelles instead of PEGylated liposomes after their concentration exceeded a critical limit [50,51]. It is found that stabilization of the lipid cores can also be measured by their CMC; that is, the concentration at which the copolymer chains start to associate themselves to form micelles [17]. It is observed that many PEG-PE compositions have CMCs in a range of 1025 M, which is at least 100-fold lower than those of conventional detergents [52]. 

## 3. Hybrid Nanomicelles with Metal Nanoparticles

Hybrid composition of nanoparticles can gain many positive features, from both mixing materials and then generating new single composites we are able to meet the requirements in applications such as labeled materials, photonic nano-devices, or chemical sensors [54]. Encapsulation of metal nanoparticles in micellar aggregates can serve many purposes: (1) improving stability [55]; (2) reducing toxicity [56]; (3) easy to be multi-functionalized; (4) improving collective properties; and (5) serving as a template for functional cavity formation. In 2005, the Taton group produced hybrid micelles depending on amphiphilic-polystyrene-block-poly(acrylicacid) (PS-PAA) and gold NPs by using the co-precipitation approach [57]. Similarly, gold nanoparticles (AU NPs) were integrated inside PS-PAA. Since hydrophilic poly(acrylic acid) (PAA) shell can stabilize the hybrid micelles and remains soluble in water, which also can be attached by using cross-linker such as carbodiimide (EDC) that effectively avoids dissociation in organic solvent.

**Scheme 4:**
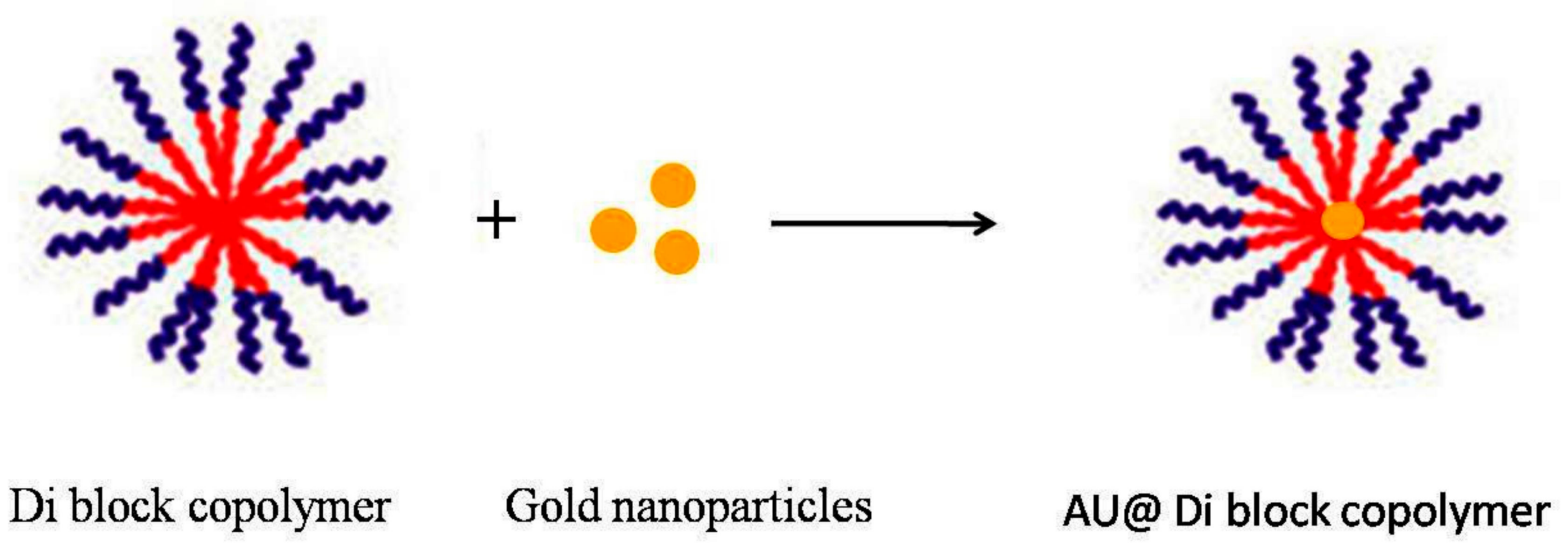
General scheme for the formation of the hybrid compound micelles.

Mantzaridis and Pispas developed a new hybrid polymeric colloidal system, where gold nanoparticles are integrated inside the micelles of a poly(styrene-*b*-2-vinyl pyridine) (PS-P2VP) di-block copolymer in toluene and then they are encapsulated again in larger micelles of another rdi-block copolymer, namely poly(isoprene-b-styrene) (PIPS). This approach could be useful in circumstances that require different outer coronas of the micelles, rather than the one that is used in the nano-reactor scheme for metal nanoparticle production (Scheme 4) [58].

## 4. Micelles Sensitive to Biological Stimuli

These micelles are formed to respond to biological stimulation, offering a great opportunity for drug delivery as controllable systems in the treatment of cancer therapy. The stimuli systems cause the micelles to answer their effect allowing drugs to be released respecting to specific external or internal stimuli, such as temperature, pH, ultrasound or enzymes, by including thermo- or pH-sensitive components or by attaching specific targeting moieties to the outer hydrophilic surface of polymeric micelles [59]. Among these stimuli, pH-sensitive polymeric micellar systems are developed, and they depend on two main strategies: pH-sensitive polymer-drug conjugates, such as hydrazone, cis-acotinyl, and acetal bonds [60]. These bonds are stable at neutral or alkaline pH but occur hydrolytic cleavage at acid pH [61].

Another strategy is to attach “titratable” groups in the copolymers, such as amines or carboxyl groups, to control micelle formation by producing physical or chemical dissociation. Hovewer pH-sensitive polymers can be protonated at pH, causing polymer rapture and drug release. Many protonated polymers have been published, such as poly(histidine) (polyHis), poly(acrylic acid) and poly-sulfonamides. PolyHis is the common pH-sensitive polymers can be used because it contains an imidazole ring endowing it with pH-dependent amphoteric properties [62]. For example, tri-block copolymer PLA-*b*-PEG-*b*-polyHis micelles were designed. Doxorubicin showed 60% release at pH 6.8 and 74% at pH 6.0, while only minimal release observed at pH 7.4 [63]. In an advanced work, new “nano-elastic” micelles have been developed. This property has been showed through the principle dissolution of polygalacturonic acid (PgA), since PgA is not soluble at the acidity of the stomach due to its hydrophobicity, while it is soluble in the pH of the colon. The strategy used is to block the PgA chain with polyacrylic acid (PAA) forming PgA-PAA composition. This property allows micelles to answer to the gastrointestinal tract pH. Therefore, they can swell at alkaline pH and shrink at acidic pH. In this case, PgA-PAA can pass gastrointestinal tract with no dissolution [64].

The modification of micelles is summarized on Table 1.

## 5. Drug-Loaded Micelles

Drugs can be encapsulated inside micelle moieties either by physical properties or chemical attachments. Drugs loaded by chemical conjugation are released by bulk degradation or surface erosion of the polymer, while drugs loaded by physical entrapment are released by diffusion. Drug release is furthermore affected by the extent of micelle moieties, resulting in slower release, leading to extended release times [65]. Doxorubicin and paclitaxel have been recently approved for clinical trials, and are good examples of micelles used to carry chemotherapies [66]. Several methods are widely used to load drugs inside micelles: (1) oil-in-water (O/W) emulsion techniques [67]; (2) water-in-oil-in-water (W/O/W) emulsion techniques [68]; (3) direct dialysis [69]; (4) co-solvent evaporation [70]; and (5) freeze-drying/lyophilization [71].

Among the abovementioned methods, O/W, direct dialysis, and co-solvent evaporation are well suited for the encapsulation of hydrophobic drugs, whereas W/O/W is often preferred for the encapsulation of more hydrophilic compounds [72]. Application of polymeric micelles for drug delivery depends on the stability of micelles at both thermodynamic and kinetic potential after their intravenous injection and dilution in the vascular compartment. Release of the drug out of its target site can be avoided completely if the structure of micelles is stable enough upon intravenous administration, and they remain as nanoparticles long enough to accumulate in sufficient concentrations at the target site [73].

## 6. Advantages and Disadvantages of Micelles

The main characteristic of micelles is the core-shell structure. Hence the hydrophobic drugs can be still stable in water, and the corona shell, causing protection for the drug by preventing elimination by the mononuclear phagocyte system (MPS) [74], which enables prolonged their blood circulation [35]. Additionally, micelles have less toxicity and can be removed by renal filtration [75]. Micelles can save and derive the water-insoluble drugs in their hydrophobic core [76,77]. The ideal micelles for delivery of hydrophobic drugs were limited for those characterized by a hydrophilic corona to stabilize and protect the hydrophobic drug. Water solubility of drugs can be increased from 10- to 500-fold if they are encapsulated inside polymeric micelle moieties [78], which enables the intravenous injection of micelle-encapsulating hydrophobic drugs. For example, although paclitaxel is a water-insoluble drug, its water solubility is significantly enhanced when it is encapsulated in a micelle [79].

Concerning disadvantages of micelles, several challenges associated with their use concern the stability of micelles in the blood stream, where critical micelle concentration could be reduced by blood dilution, and the encapsulated drugs can leak out of the polymer assembly, minimizing the drug circulation half-life [80,81]. In order to overcome these issues, physical-chemical properties of micelles were addressed to improve stability and loaded drug efficiency inside micelles, one of the physical interactions between micelle moieties and the drug is a pi-stacking (π-π stacking) interaction. It is applied to be the subject of doxorubicin encapsulation into PEG-*b*-poly(α,β-aspartic acid) with the poly(α,β-aspartic acid) block co polymers [82]. Free radical polymerization was used upon assembly of modification of PEG-*b*-PLA with methacrylic acid anhydride, resulting in good stability of micelles’ structure [83]. Another notable strategy is to integrate drug attached polymers into lipids to prevent leakage out of the layer capsules [54]. Recently, micelles entrapped into CaCO_3_ crystals were coated by alternate adsorption of several layers forming, after core removal, several layers around the micelles’ structure [84].

## 7. Applications of Micelles in Cancer Therapy

Micelles that were being characterized by their small size have received scientific interest, since their diameters enables them to penetrate the vasculature of tumors effectively [83]. For instance, paclitaxel encapsulated inside moieties of polymeric micelles showed high drug capacity and good efficiency in patients with advanced malignancies [84,85,86], metastatic breast cancer [87] and advanced non-small lung cancer [88]. Similarly, paclitaxel, pluronic polymer-bound doxorubicin (SP1049C) [89], and NK911, a micelle-encapsulated doxorubicin [90], were used extensively for cancer treatment. The hydrophobic core of micelles designed from lipid conjugated PEG can be occupied by several insoluble drugs, such as paclitaxel, tamoxifen, porphyrin [91], camptothecin [92], and vitamin K3 [93]. In aqueous solution, adriamycin–attached into (PEG-P[Asp(ADR)]) exhibits antitumor activity in vivo [94].

Curcumin conjugated with PEG exhibited good cytotoxicity against several human cancer cell lines compared to free curcumin, such as breast [95], colon [96], prostate [97], kidney [98], liver [99] lymphoid and myeloid tissues [100], and melanoma [101]. Curcumin attached with Beta-thioester bonds can be selectively released by glutathione and esterase [102]. Hanafy et al. succeeded in loading glycolysis inhibitor (Bromopyruvic acid) inside chitosan-oleic acid nano-composite. The structure exhibited good cytotoxicity effects on hepatocellular carcinoma (HLF) cell line [53]. Also, micelles containing photosensitizing agent have been used in treatment of murine lewis lung carcinoma [103].

Micelles-foliate used as targeting therapy has shown significant effects against ovarian carcinoma cells compared to non-targeted micelles [104,105]. In clinical trials, NK911 and SP1049C [104] and NK105 and NC6004 are currently passed to Phases I and II [106,107]. The hydrophobic-hydrophilic regimens of micelles may offer several advantages for cancer therapy and drug delivery systems, including: increasing their capacity for water-insoluble drugs, resulting in mostly increased drug solubility; raising their drug accumulation inside the cancer site; prolonging their drug time circulation inside the blood stream; and their corona allowing micelles not react with biological components and then not be recognized by permeability and retention. Micelles can be designed to be used in several medical applications. Various micelle structures have been used as drug delivery system to possible penetrate solid tumors such as *N*-(2-hydroxypropyl) methacrylamide (HPMA) copolymers covalently conjugating doxorubicin via enzymatically cleavable glycyl–phenylalanyl–leucyl–glycine spacer [108,109]. Similar for this structure, paclitaxel was conjugated poly(glutamic acid) [110,111] and SN-38 was integrated into PEG-*b*-poly(l-glutamic acid) [112].

Micelles used as carriers for cancer therapies were summarized in Table 2. 

## 8. Conclusions

Micelles have received great attention as an interesting drug delivery system, due to their simple fabrication, their capacity to be loaded with a wide variety of insoluble drugs, and the possibility to develop and improve their moieties. Micelles’ formation enables them to be used in wide medical applications. They can be fabricated simply by blocking copolymers, forming various shapes of micelle, or can be trapped by metal nanoparticles, or can be doped by responsive polymers to respond the biological stimuli. Micelles attached by biological molecules, such as lipids or proteins, are considered valuable and applicable to use as drug delivery systems.

## Figures and Tables

**Table 1:** Micelles modification summary.

Micelles	Formulation
Lipid micelle	Phospholipid or cholesterol
Polymeric micelles	Polymers having hydrophobic and hydrophilic properties
Hybrid polymeric lipids micelles	Polymers integrated into lipids
Hybrid micelles with metal nanoparticles	Micelles assembled with gold, silver or iron oxide nanoparticles
Micelles coated by layer by layer technique	Micelles incorporated into calcium carbonate and coated by polymers
Stimuli-responsive micelles	Micelles doped with stimuli such as pH-sensitive components

**Table 2:** Micelles as carriers for cancer therapies (reproduced from ref [81]) with permission, (Copyright Elsevier 2014).

Name	Drug	Block Copolymer	Drug Loading (%w Drug/w Polymer)	Size (nm)	Company	Indication
NK105	Paclitaxel	PEG-*b*-poly(α,β-aspartic acid)	23	85	Nippon Kayaku, Co.	Gastric cancer/Breast cancer
NK012	SN-38	PEG-*b*-poly(l-glutamic acid)	20	20	Nippon Kayaku, Co.	Triple negative breast cancer
NK911	Doxorubicin	PEG-*b*-poly(α,β-aspartic acid)	17	40	Nippon Kayaku, Co.	Various solid tumors
NC-6004	Cisplatin	PEG-*b*-poly(l-glutamic acid)	30	20	Nanocarrier, Co.	Pancreatic cancer
NC-4016	Oxaliplatin	PEG-*b*-poly(l-glutamic acid)	30	30	Nanocarrier, Co.	Various solid tumors
NC-6300	Epirubicin	PEG-*b*-poly(aspartate-hydrazone)	20	60	Nanocarrier, Co.	Various solid tumors
siRNA micelles	siRNA	PEG-*b*-polycations	Various	40–60	Nanocarrier, Co.	–
